# Protein Quakes in Redox Metalloenzymes: Clues to Molecular Enzyme Conductivity Triggered by Binding of Small Substrate Molecules

**DOI:** 10.1002/open.202400190

**Published:** 2024-10-30

**Authors:** Henrik Bohr, Irene Shim, Jens Ulstrup, Xinxin Xiao

**Affiliations:** ^1^ Department of Chemical Engineering Technical University of Denmark Building 229, Kemitorvet DK-2800 Kgs. Lyngby Denmark; ^2^ Department of Chemistry Technical University of Denmark,Building 207, Kemitorvet DK-2800 Kgs. Lyngby Denmark; ^3^ Department of Chemistry and Bioscience Aalborg University DK-9220 Aalborg, Denmark

**Keywords:** Two-centre copper nitrite reductase, intramolecular electron transfer, *ab initio* electronic stucture calculations, electrochemical STM/AFM, Frontier orbitals

## Abstract

Multicentre redox metalloproteins undergo conformational changes on electrochemical surfaces, or on enzyme substrate binding. The two‐centre copper enzymes, laccase (Type I and TypeII/III Cu) and nitrite reductase (CuNIR) (Type I and Type II Cu) are examples. With some exceptions, these enzymes show no non‐turnover voltammetry on Au(111)‐surfaces modified by thiol based self‐assembled molecular monolayers, but dioxygen or nitrite substrate triggers strong electrocatalytic signals. Scanning tunnelling microscopy also shows high conductivity only when dioxygen or nitrite is present. Atomic force microscopy shows constant CuNIR height but pronounced structural expansion in the electrocatalytic range on nitrite binding. We have recently offered a rationale, based on *ab initio* quantum chemical studies of water/nitrite substitution in a 740‐atom CuNIR fragment. Presently we provide much more detailed structural assignment mapped to single‐residue resolution. NO_2_^−^‐binding induces ***both*** a 2 Å Cu−Cu distance increase, ***and*** pronounced frontier orbital delocalization strongly facilitating ET between the Cu regions. The conformational changes transmit from the catalytic Type II centre to the electron inlet Type I centre, via the His129‐Cys130 ligands, and via Type I–Cys130 or Type I‐His129 ending at Type II Asp92. The ET patterns are reflected in different atomic Mulliken charges in the water and nitrite CuNIR fragment.

## Graphical abstract

Metalloenzyme conformational changes accompany electrocatalytic action. Two‐centre copper nitrite reductases (CuNIRs) are examples, mostly without non‐turnover voltammetry on Au(111)‐surfaces, while nitrite substrate triggers electrocatalysis, CuNIR conductivity, and enzyme structural expansion. We offer a quantum chemical rationale. NO_2_^−^ induces both 2 Å Cu−Cu distance increase in 740‐atom CuNIR fragments, and frontier orbital conversion from localized to delocalization between the Cu regions, triggering electronic conductivity.

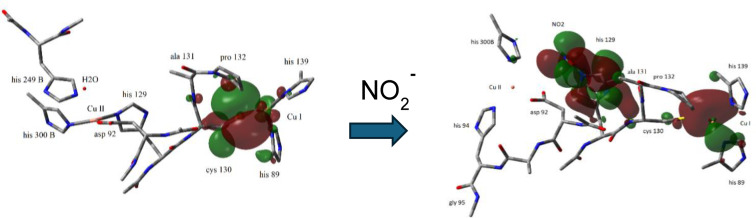

## 1 Introduction

Conformational triggering, or cooperative nuclear conformational effects in multi‐centre redox metalloproteins can accompany electron transfer (ET), enzyme substrate binding, and metalloprotein binding to electrochemical electrode surfaces.[^1^, ^2^, ^3^, ^4^, ^5^] Conformational triggering can lead to drastic changes in ET rates,^[6]^ spectral properties,^[7]^ substrate binding constants as in hemoglobin oxygenation,^[2]^ and enzyme activity.[^3^, ^5^, ^6^] Conformationally triggered activity change has been identified e. g. as opening of intramolecular ET channels,[^3^, ^5^] possibly accompanied by proton coupled ET (PCET).[^8^, ^9^] New perspectives in metalloprotein bioelectrochemistry are scanning tunnelling and atomic force microscopy directly in aqueous biological media under electrochemical potential control (in situ or *in operando* STM and AFM)‐[^10^, ^11^, ^12^, ^13^, ^15^] *In situ* STM/AFM offer novel clues to conformationally triggered metalloprotein activity, right down to the single metalloprotein molecular level, opening for a quite new bioelectrochemistry.

***Two‐centre*** metalloenzymes/‐proteins are prototype multicenter proteins, displaying conspicuous conformational cooperative effects, at the same time being simple enough that detailed kinetic resolution is within reach.[^3^, ^6^, ^7^] Proteins with larger numbers of centres, such as the four‐centre proteins hemoglobin^[2]^ and cytochrome *c*_3_^1^ are perhaps engaged in wider ranging conformational changes but are too complex for complete molecular scale mapping of ET or ligand binding (for a discussion, see e. g.).[^6^, ^7^]

Three particular two‐centre metalloenzymes/‐proteins illustrate conformationally triggered enzyme activity or electron transfer on enzyme substrate binding, or protein binding to electrochemical surfaces. One is the di‐heme ET protein cytochrome *c*_4_ (*Pseudomonas stutzeri*).[^6^, ^12^] Intramolecular ET between the two heme groups is not part of the ET network between cyt *c*_4_ and external reaction partners in homogeneous solution, but cyt *c*_4_ immobilization on mercaptodecanoic acid modified Au(111)‐electrode surfaces opens fast (sub‐ms) intramolecular ET between the two heme groups. Spectral differences between the two heme groups are too small for conclusive spectral distinction.[^6^, ^10^] This differs from two‐centre desulfoferrodoxin (*Desulfovibrio desulfuricans*), for which Fe‐coordination, UV‐vis/MCD spectral properties, and redox potentials can all be clearly distinguished.^[7]^

Human sulfite oxidase (hSO) is a second case for combined single‐crystal voltammetry and single‐molecule *in situ* STM of a conformationally triggered metalloenzyme.^[16]^ Dimeric two‐domain hSO accommodates a catalytic molybdenum cofactor (Moco) and a cyt *b*_5_ heme domain. The latter is separated from the Moco domain by a polypeptide loop, which controls switching between a “closed”, catalytically inactive conformation and an open conformation that triggers ET between the Moco cofactor and the electrode ***via*** the heme domain. Cellobiose dehydrogenase (*Myriococcum thermophilum)* operates by a similar mechanism, i. e. ET from the catalytic centre (flavine adenine dinucleotide, FAD) to the electron acceptor (molecular dioxygen or the electrode) via a conformationally mobile heme group domain.^[15]^

**Figure 1:**
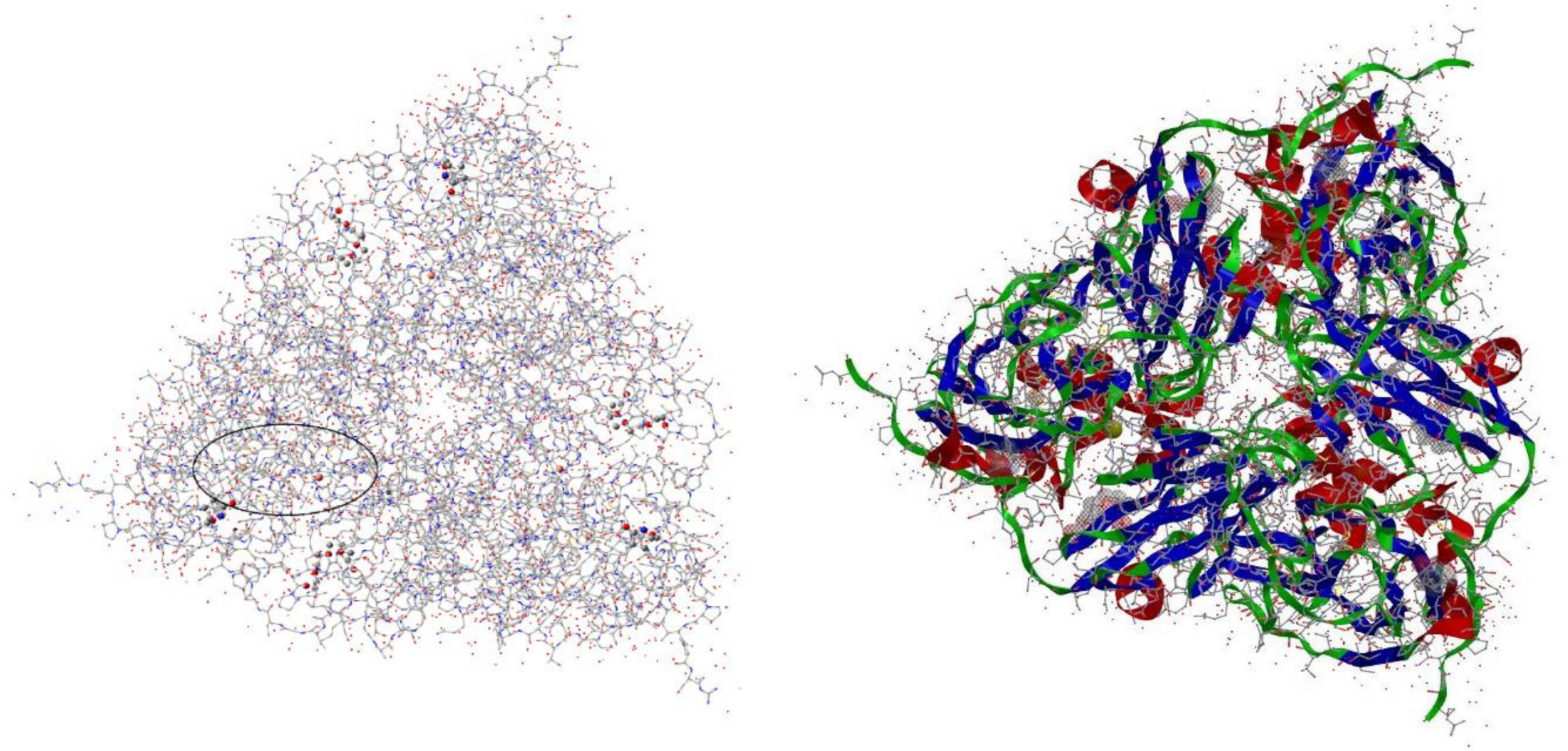
Left: *A. xylosoxidans* CuNIR trimer structure (**PDB 1OE1**) viewed along the trigonal axis to illustrate the 740 atom protein fragment carved out for detailed *ab initio* Hartree‐Fock computations. The Type I/Type II units are indicated by orange spheres. The ellipsoidal frame shows the location of our 740 atom target model fragment in the overall protein structure. Right: Corresponding ribbon view of the **PDB 1OE1** protein structure.

A third case is the trimeric two‐centre blue copper enzyme nitrite reductase, CuNIR (*Achromobacter xylosoxidans*, PDB 1OE1), Figure 1. CuNIR is central in the biological nitrogen cycle, where it catalyzes one‐electron reduction (stoichiometrically a proton coupled electron transfer process, PCET) of the nitrite ion substrate to NO.[^16^, ^17^, ^18^, ^19^, ^20^, ^21^]
(1)

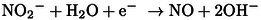




*A. xylosoxidans* CuNIR is the target metalloenzyme in our present theoretical and computational study of the binding of the substrate NO_2_^−^ to a model CuNIR structural fragment.[^3^, ^5^] The major conformational and electronic changes found, triggered by the NO_2_^−^ binding hold clues to both CuNIR electrocatalysis and single‐molecule conductivity patterns.

## 2 *A. Xylosoxidans* CuNIR and CuNIR Fragments – An Overview

Figure 1, left shows an overarching view of the trimeric *A. xylosoxidans* CuNIR structure (PDB 1OE1). The oval region shown is a 740 atom enzyme fragment chosen for our detailed investigations of the molecular and electronic structural changes that accompany replacement of a Type II bound water molecule by the substrate NO_2_^−^. As a comparison, Figure 1, right shows a more common ribbon structural view of the same molecule.

Carving out a small fragment of the large protein is obviously a limitation but needed by our choice of the accurate *ab initio* Hartree‐Fock (HF) method. Larger parts of the protein including protein binding to solid surfaces can be addressed, but only by classical molecular dynamics (MD) approaches and, to an extent Density Functional Theory (DFT). Our limited‐size but self‐consistent and in principle expandable accurate *ab initio* model, however, captures essential features of H_2_O→NO_2_^−^ substitution that accord with our reported experimental data from single‐crystal voltammetry, and electrochemical *in situ* STM/AFM, including a frame for the “protein quake” notion. Methodological challenges associated with quantum mechanical computational approaches to large protein molecules are discussed in detail by E.I. Solomon and associates, with particular reference to blue oxidases.[^22^, ^23^]

**Figure 2:**
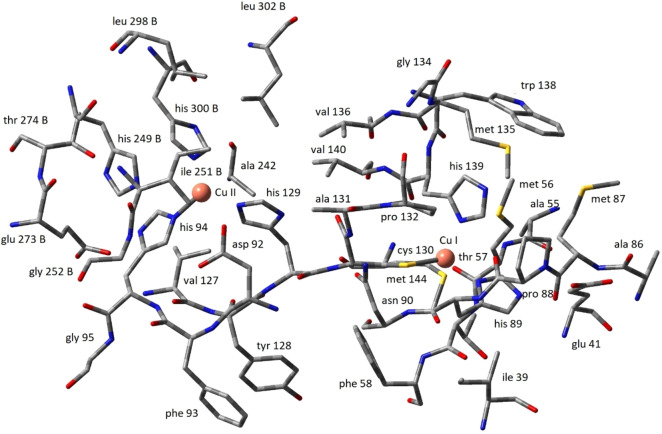
Structural details of the ligand spheres and connecting residues around the two Cu‐centres in the 740 atom CuNIR (PDB 1OE1) native fragment.

Figure 2 shows details of the 740 atom fragment carved out of the experimental crystallographic molecular structure (**PDB 1OE1**). The Figure shows the details of the ligands around the two Cu atoms in their “native” form. Figures 4 and 5 show the ***computationally converged*** 740 atom fragment structures. The direct through‐bond link between the Type I and Type II centres are visible, but better illustrated by the CuNIR fragment structures shown in Figures 6, 7, 8, 9. The distance between the Cu I and Cu II atoms in the crystal structure (PDB 1OE1) is 12.43 Å. The ligand sphere around the electron inlet Type I centre is constituted by His89 (distance 2.02 Å from Cu I), Cys130 (2.20 Å), His139 (2.03 Å) and Met144 (distance Cu−S 2.45 Å). The ligand sphere around the catalytic Type II centre consists of Asp92 (3.54 Å), His94 (1.96 Å), His129 (2.00 Å), and His300 (2.00 Å). Notably the Cu‐centres are directly covalently linked via the Cys130 ligand of the Type I centre and the Type II His129 ligand.

Blue Cu‐enzymes including CuNIR, themselves often show weak or no non‐turnover voltammetry.[^5^, ^24^, ^25^, ^26^] The voltammetric silence suggests either that the intramolecular covalent superexchange ET channel linking the two centres is closed, or that the electronic contact to the SAM modified electrodes is hampered in this “resting state”. The latter depends, however, sensitively on the particular hydrophilic or hydrophobic nature as well as on the packing modes of the SAMs, mapped in some detail for *A. xylosoxidans* CuNIR on Au(111)‐electrode surfaces modified by a variety of thiol based SAMs.[^2^, ^24^] U. Contaldo and associates in fact reported non‐turnover voltammetry of a different CuNIR (*Alcaligenes faecalis*) linked hydrophobically to multi‐wall carbon nanotube surfaces.^[27]^ It is notable that *in situ* AFM points clearly to dense CuNIR monolayer coverage on cysteamine SAM modified Au(111)‐electrode surfaces, both in the presence and absence of nitrite substrate.^[28]^ The absence of non‐turnover voltammetric signals cannot therefore be caused by protein detachment from the electrode surface.

Binding of NO_2_^−^, however, induces strong electrocatalytic turnover signals at pH 6.0. This pH value is higher than the pH value where maximum enzyme activity is reported and was chosen so that enzyme catalyzed NO_2_^−^ conversion to NO is dominated by reaction with H_2_O, Equation (1) and acid catalysis minimized.[^5^, ^20^] Onset of nitrite triggered electrocatalytic signals likely indicates both that an intramolecular ET channel between the two copper centres has opened, and that protein surface reorientation may have facilitated electrode‐protein contact.

**Figure 3:**
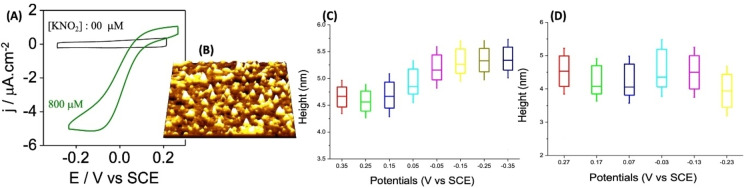
(A) Cyclic voltammograms of *A. xylosoxidans* CuNIR (PDB 1OE1; 5 mM acetate buffer, pH 6.0. (B) *In situ* STM of *A. xylosoxidans* CuNIR (PDB 1OE1); 0.2 mM KNO_2_; 5 mM acetate buffer, pH 6.0. Tunnelling current 0.1 nA., electrode potential +0.38 V (SCE), bias voltage −1.10 V; “effective” potential at the protein centre ≈−0.17 V (SCE). (C) *In situ* AFM in the presence of 110 μM nitrite substrate and (D) absence of nitrite substrate; 10 mM acetate buffer, pH 6.0. Data from.[^5^, ^11^, ^24^, ^28^]

Intramolecular ET is further illuminated by *in situ* STM with strong single‐molecule contrasts, i. e. high molecular conductivity when substrate nitrite is present but not in the absence of nitrite. Further, single‐molecule *in situ* AFM discloses substantial conformational changes in the form of ≈1 nm expansion (“swelling”) of CuNIR in direct nitrite reducing action on a cysteamine modified Au(111)‐electrode surface, but no change in the absence of nitrite, Figure 3.^[28]^ Such observations are intriguing; enzyme “swelling” might otherwise be expected to attenuate or close intramolecular ET but this appears to be compensated by improved intramolecular electronic overlap and perhaps improved electronic contact between the CuNIR and the cysteamine SAM modified Au(111)‐electrode surface. The conformational change further appears to depend reversibly on the electrochemical potential, i. e. expansion and contraction, when the potential is scanned in opposite directions.^[29]^ “Swelling” is not a CuNIR feature in the crystalline state[^17^, ^18^, ^19^, ^20^, ^22^, ^23^] which, however, is not the natural medium for enzyme function.[^3^, ^22^, ^23^]

## 3 Nitrite Triggered Conformational Enzyme “Quakes” – A Computational Overview

Following our previous study,^[3]^ we provide here a more complete mapping of the conformational changes that accompany the H_2_O→NO_2_^−^ substitution, now resolved to the level of the individual amino acid residues, both in the ligand spheres of the Type I and Type II Cu centres and in between. We also address changes in all the Mulliken atomic population charges on the substitution. Conformational reorganization spreads through the whole protein fragment warranting the collective “protein quake‐like” conformational notion.

We follow our recently reported computational procedure to address the intriguing pattern summarized in Figure 3.[^3^, ^5^, ^24^, ^28^] Briefly, we first carved a 3.3 nm 740 atom fragment out of the ***“experimental”*** CuNIR monomer subunit. The fragment contains the two Cu‐centres with ligands and adjacent amino acid residues, viewed in detail in Figure 2, altogether 37 residues. Valences ending in carbonyl groups were saturated with N‐methyl groups, those ending with Nitrogen with acetyl groups. The Type I/Type II structural unit was addressed fully quantum mechanically, focused on Type II H_2_O→NO_2_^−^ substitution, with conformational changes extending throughout the protein fragment. We used the *ab initio* Hartree‐Fock self‐consistent field method (HF‐SCF) in the Roothaan formulation,^[30]^ along with the Born‐Oppenheimer approximation. For details of the HF/6‐31G* approach, see for example.[^30^, ^31^]

**Figure 4:**
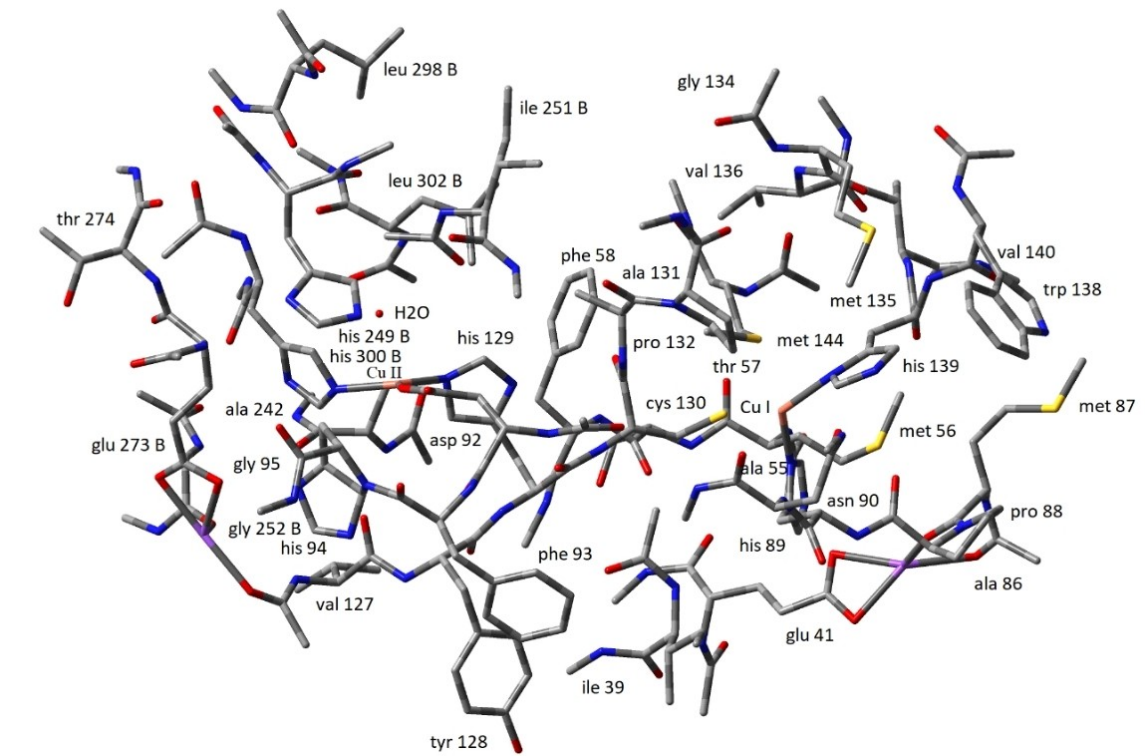
Structural details of the ligand spheres and connecting residues around the two Cu‐centres in the 740 atom CuNIR fragment containing a H_2_O molecule. The structure **is** HF‐SCF optimized.

**Figure 5:**
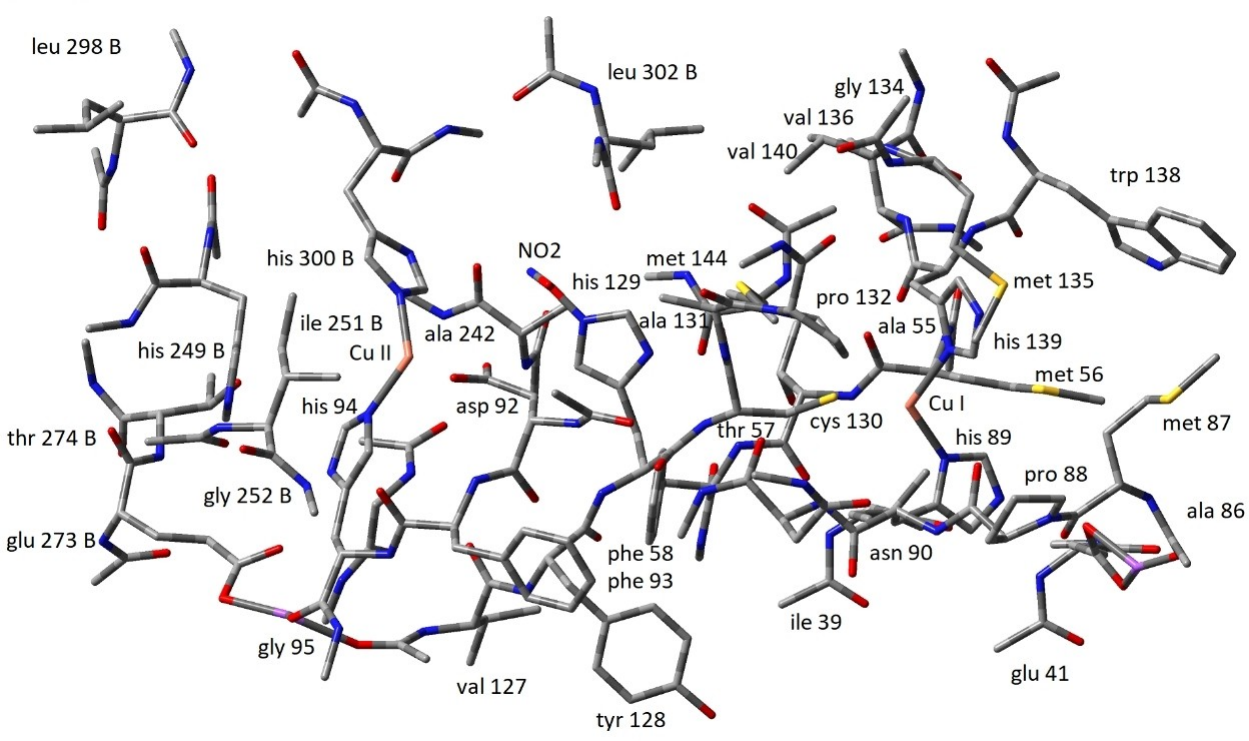
Structural details of the ligand spheres and connecting residues around the two Cu‐centres in the 740 atom CuNIR fragment containing a nitrite ion. The structure **is** HF‐SCF optimized.

Wave functions were expressed as Slater determinants, and molecular orbitals (MOs) as linear combinations of atomic orbitals (LCAOs) in 6–31G* Gaussian type basis sets. The calculations were carried out using Gaussian 16.^[31]^ A H_2_O molecule was added to the Type II Cu centre, with the distance between Type II Cu and the O atom of H_2_O set to 2.0 Å. Likewise a NO_2_^−^ ion with a 2.0 Å Type II Cu−N atom distance. The fragment structures were optimized as noted. The calculations converged, when the maximum displacement was less than 0.0018 a.u. (1 a.u.=0.53 Å) and the maximum force less than 0.00045 a.u. (1 a.u.=82 nN). The converged ***computed*** structures are shown in Figures 4 and 5. These can in principle be compared with the ***“experimental”*** structure shown in Figure 2 by inspection, but the many details in these views would blur visual distinction of the differences. Instead we addressed the differences by a following round of computations described below.

The choice is prompted by HF/6‐31G* being an *ab initio* method, and that the results can be improved systematically by including electronic correlation effects with increasing detail. Experimental interatomic distances in organic molecules are well reproduced in HF/6‐31G* calculations, typically off by only 0.01–0.02 Å. Mean absolute errors of energies between conformational isomers are around 2.5 kJ/mol, i. e. close to room temperature thermal energy *k*_B_ *T* (*T* the temperature and *k*_B_ Boltzmann's constant). The large numbers of local potential energy minima of a N‐atom molecule with 3 N−6 degrees of freedom means that HF or any other calculation based on the variational principle give minima close to the starting point. It is, however, difficult to include extra solvent molecules other than ligand molecules such as bound water and nitrite in CuNIR.

The H_2_O molecule added induces some conformational changes in the optimization process. The distance between Cu I and Cu II in the optimized structure is 12.07 Å, close to the original crystal distance of 12.43 Å. The coordination around the Cu centers converges as: Type II Cu coordinated to His300 (distance 2.08 Å), His129 (2.04 Å), and to the O atoms of Asp 92 (2.01 Å and 4.16 Å, i. e. essentially coordination via a single O‐atom). In the converged structure the Cu−O distance to H_2_O is 3.73 Å. The distance between Type II Cu and His94 changes from 1.96 Å in the crystal to 5.53 Å in the converged structure, i. e. essentially dissociation of the Type II/His94 bond.

The Type I Cu center has the same coordination in the converged structure as in the crystal, with slightly larger Cu‐ligand distances. The Cu‐His89 distance is 2.08 Å, the Cu‐His 139 distance 2.10 Å, and the Cu‐Cys130 distance 2.38 Å. The distance between Type I Cu and the S atom of Met144 has increased to 3.44 Å from 2.45 Å in the crystal.

When a nitrite ion is added to the 740 atom fragment, the Type II Cu remains tetra‐coordinated, but with the distances now: His300 (distance 2.08 Å), His 94 (2.05 Å), and to the two O atoms of Asp92 with distances 2.27 Å and 2.24 Å. The 2.00 Å distance of His129 coordination to Type II Cu in the crystal has changed to 5.33 Å. Both His129 and Asp92 thus undergo major conformational changes. The Type II Cu‐His129 link has dissociated in the nitrite bound state, possibly triggered by the tight NO_2_^−^ binding to His129 with an O−N distance of 1.34 Å. Asp92 undergoes a reverse change, from loosely bound (2.01/4.16 Å) in the H_2_O fragment to tightly bound (2.27/2.24 Å) in NO_2_^−^ bound form, respectively. This could be a likely key origin of the overall conformational and electronic changes on H_2_O→NO_2_^−^ substitution.

Type I Cu in the converged structure with NO_2_^−^ is coordinated to His89 (distance 2.03 Å), His 139 (2.14 Å), and Cys130 (2.31 Å). Met144 is coordinated to Type I Cu with distance 2.45 Å in the crystal. This distance has increased to 3.44 Å In the H_2_O fragment, and to 7.56 Å in the nitrite fragment, implying also here significant conformational relaxation on H_2_O→NO_2_^−^ substitution. The distance between the Cu atoms in the converged structure is 14.46 Å. Structural expansion, or “swelling” by 2.39 Å on H_2_O→NO_2_^−^ substitution has thus occurred, triggered by the major conformational changes in the ligand spheres of both Cu‐centres (His129 and Asp92 at Type II Cu, Met144 at Type I Cu).

**Figure 6:**
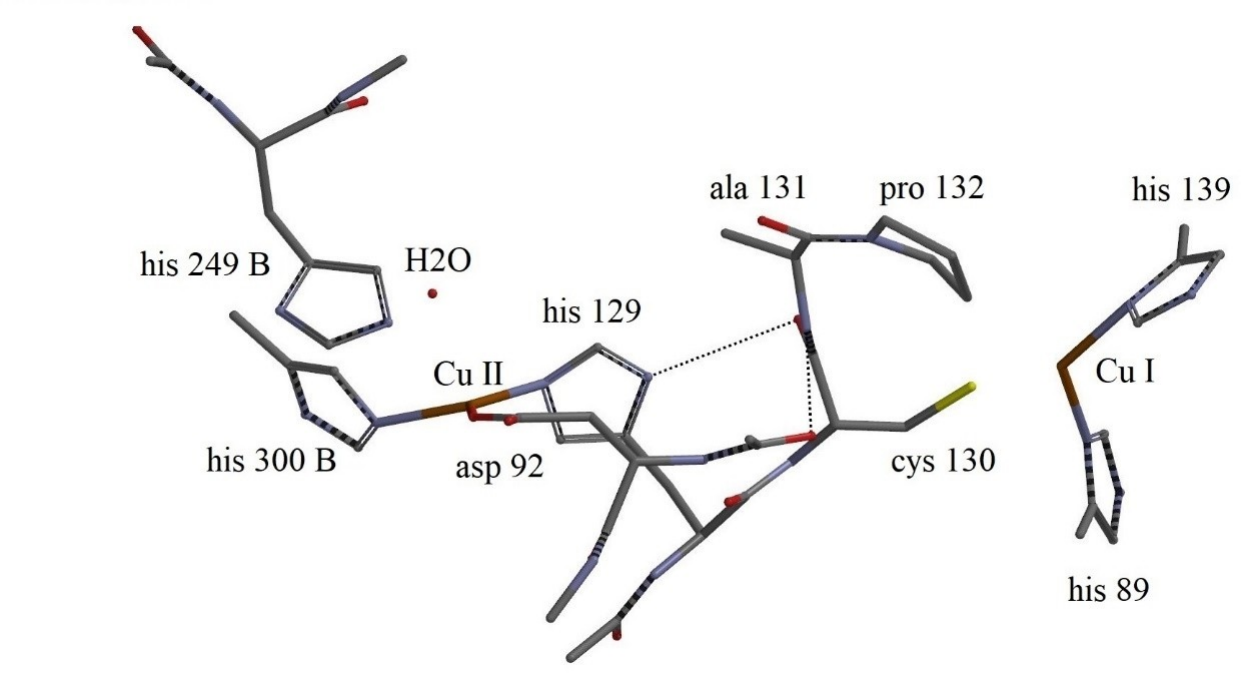
Conformational structure of the 150 atom molecular CuNIR H_2_O fragment structure with bound water. Water is bound via a H‐bond to the His129 (2.39 Å) Type II Cu ligand. Amino acid numbering, and location of the Type I and Type II Cu‐centres indicated. Central hydrogen bonds indicated by dashed lines.

**Figure 7:**
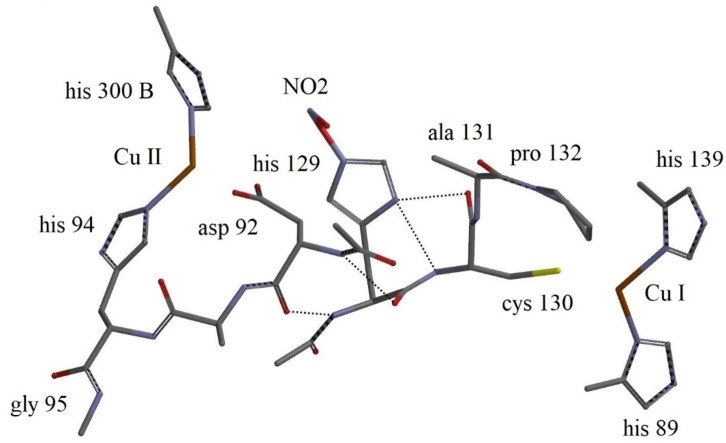
Conformational 148 atom NO_2_^−^ fragment structure. Amino acid numbering, location of the Type I and Type II Cu‐centres, and the nitrite binding indicated. Central hydrogen bonds indicated by dashed lines.

In order to disentangle further the conformational reorganization and electronic changes triggered by H_2_O→NO_2_^−^ substitution, the converged 740 atom structures were stripped of the outer amino acids leaving solely the essential fragments with the core Type I/Type II Cu centres, their ligands, and Type II bound H_2_O or NO_2_^−^. The final H_2_O fragment contained 150 atoms, Figure 6, the NO_2_^−^ fragment 148 atoms, Figure 7. Comparison between Figures 4 and identifies the amino acids excluded. Likewise, comparison of Figures 5 and 7 identifies the amino acids excluded from the nitrite fragment. The branches extending from His300, His 139, and His89 in the H_2_O fragment and the branches in the nitrite fragment extending from His300, His139, and His89 were all replaced by methyl groups. Single‐point calculations with fixed geometries on these reduced fragments were carried out.

Figures 6 and 7 also illustrate central hydrogen bonds, indicated by dashed lines. The H_2_O fragment has two hydrogen bonds, between His129 and Cys130 with distance 2.26 Å, and between Asp92 and Cys130, with distance 2.31 Å. There is also a 2.39 Å hydrogen bond between His129 and H_2_O (not shown). Four hydrogen bonds dominate the NO_2_^−^ fragment, i. e. two between His129 and Cys130 (2.10 and 2.06 Å), and two more, between His129 and Asp92 (2.31 and 2.41 Å).

It might ***appear*** from the fragment images that Pro132 could be coordinated to Type I Cu, but this is not the case. The Type I Cu/Pro132 distance in the H_2_O fragment is 6.55 Å, the distance between the C atom of Pro132 closest to Type I Cu 5.60 Å. The corresponding distances in the nitrite fragment are 6.41 Å and 4.92 Å, in all cases far too large for electronic superexchange.

## 4 Conformationally Opened Electronic Coupling on H_2_O→NO_2_^−^ Substitution

Conformational reorganization on H_2_O→NO_2_^−^ substitution, involving the protein framework between the Type I and Type II Cu‐centres are clearly distinguished in Figures 6 and 7. In addition to the Cys130/His129 through‐bond contact, a superexchange ET pathway, viz. Cys130/His129/Asp92, possibly with additional H‐bond mediated electron exchange with Ala131 may also contribute to the overall ET pathway. The H‐bond distance between Ala131 and Asp92 is thus 3.56 Å, but 2.31 Å in the H_2_O fragment.

**Figure 8:**
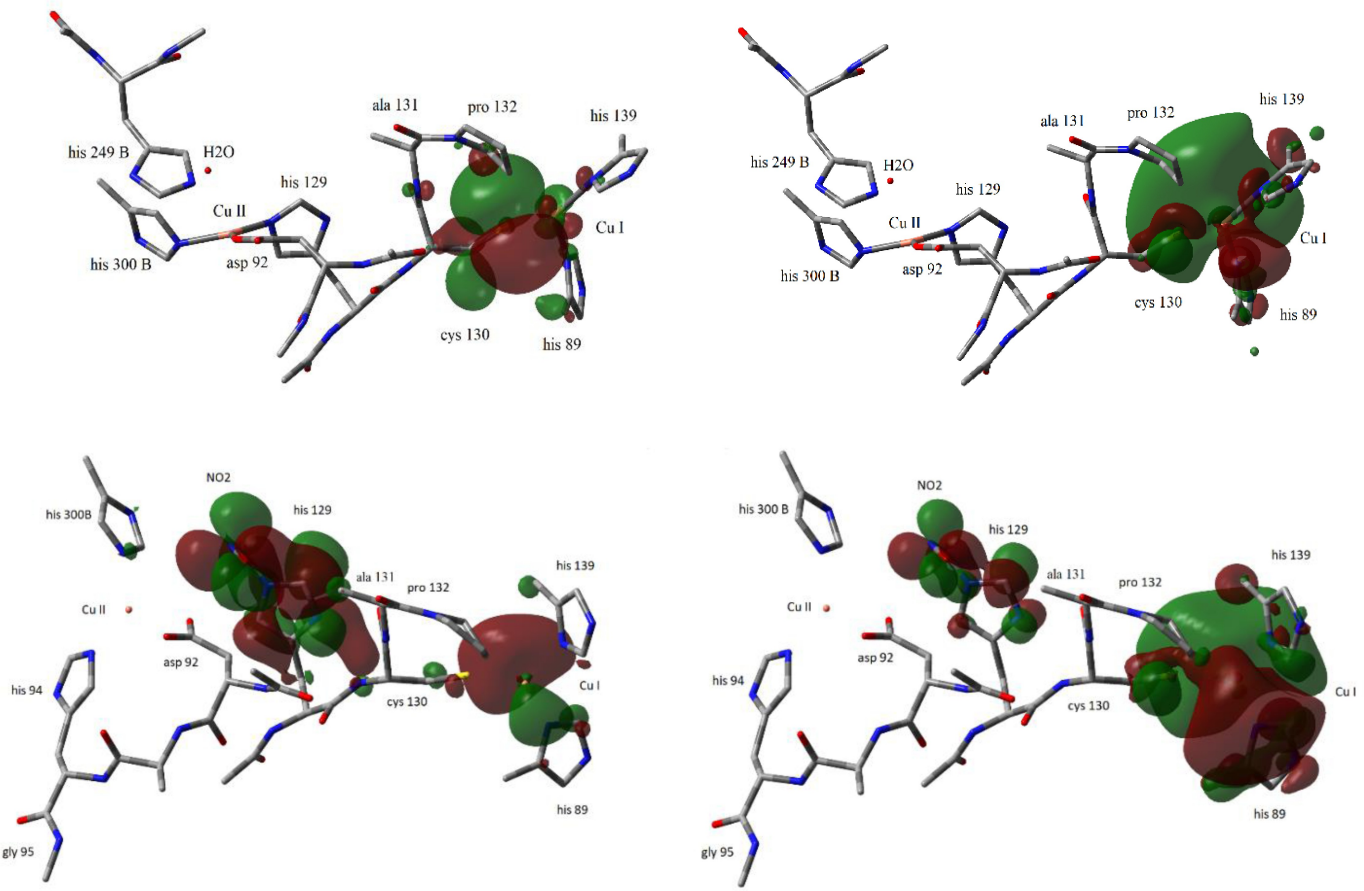
Top: HOMO (left) and LUMO (right) isosurfaces of the 150 atom H_2_O fragment. The frontier orbital location of the Type I and Type II centres largely localized on the Type I centre. The colours show the phases of the wave functions, either 1 or −1. Bottom: HOMO (left) and LUMO (right) isosurfaces of the 148 atom nitrite fragment. Colours as above. Iso values were chosen as 0.015 (electrons/a.u.^3^)^1/2^

Substitution of water by nitrite at the Type II centre was noted to cause a>2 Å Cu−Cu distance increase, or structural enzyme “swelling”. Structural “expansion” itself might attenuate or close the Type I/Type II electronic conductivity, but a drastic delocalization of the frontier MOs, Figure 8 is a second key observation.

Figure 8 shows the HOMO and LUMO iso‐surfaces of the H_2_O fragment (top) and the NO_2_^−^ fragment (bottom). Both orbitals of the water fragment are localized at the Type I centre. In our previous report^[3]^ all the orbitals LUMO to HOMO‐4 were displayed. This totality of MOs showed a distribution of electron densities between the Type I and Type II centres but with conspicuous through‐space regions in between. In either view ET between the Type I and Type II Cu centres is therefore highly unfavourable.

Before optimization the nitrite fragment HOMO is localized around Type II Cu and the LUMO around the Type I centre, i. e. far apart in space (not shown). Both the HOMO and the LUMO are, however, significantly delocalized in space during the optimization process, Figure 8, bottom. It is further noted that the major HOMO density is around Type II Cu and the major LUMO density around Type I Cu. This could suggest that ET from HOMO to LUMO, or from Type II Cu to Type I Cu dominates. The electronic density difference is, however, small and could be reversed in an expanded model that includes elements of protein and solvent dynamics, but electron density delocalization would apply equally to ET in both directions.

The HOMO and LUMO are thus significantly delocalized over the whole region between the two centres in the NO_2_^−^ fragment, even to the extent that new residues such as Ala131 may become involved in the Type I/Type II electronic coupling. Such a drastic electronic reorganization would offer much improved superexchange conductivity of the NO_2_^−^ fragment compared with the spatially localized orbitals in the H_2_O fragment. This is supported by the structural enzyme expansion that possibly leads to improved CuNIR contact to electrochemical electrode surfaces. Our computed combined conformational protein structural change that leads to drastic HOMO/LUMO alignment prompts association with the notion of “protein quakes” introduced by Frauenfelder and associates.^[32]^ “Protein quakes” in CuNIR are conspicuous in the Type II coordination sphere, the translational shift of the Type I and Type II Cu centres, and by the drastic electronic reorganization, that transmits through the whole Cu‐enzyme fragment.

A notable final observation is that neither H_2_O nor NO_2_^−^ in the CuNIR fragment coordinate directly to the Type II Cu centre. H_2_O is bound via H‐bonds to the N‐donor atoms of both Type II His129 (2.39 Å) and His300 (2.78 Å) ligands. Four hydrogen bonds dominate the NO_2_^−^ fragment, i. e. two between His129 and Cys130 (2.10 and 2.06 Å), and two more, between His129 and Asp92 (2.31 and 2.41 Å).

NO_2_^−^ engages in 4.3 Å non‐covalent bonding to Type II Cu. This binding mode differs from the crystallographic binding, in which NO_2_^−^ is bound to Type II Cu in μ‐coordination via the two oxygen atoms (CuNIR from *Rhodobacter sphaeroides* PDB 1WA1 and 1WA2).^[22]^ As noted, such differences are hardly surprising, as neither the crystalline state nor the CuNIR model fragments correspond to the enzyme catalytic or bioelectrochemical environment.^[24]^ The Type II Cu configuration changes considerably during optimization, ending up coordinating to His94, His300, and the carboxylate group of Asp92. NO_2_^−^ interacts strongly with His129 which is displaced from Type II Cu on H_2_O→NO_2_^−^ substitution (2.04→5.33 Å) and perhaps helps to bridge the electronic contact between His129 and Type II Cu, as the non‐covalent nitrite O atom distances from the N atom of His129 are only 1.34 and 2.49 Å, respectively. Strong non‐covalent, van der Waals‐like binding is known also in other contexts.^[33]^

## 5 Electrostatic Charge Redistribution on Nitrite Binding

**Figure 9:**
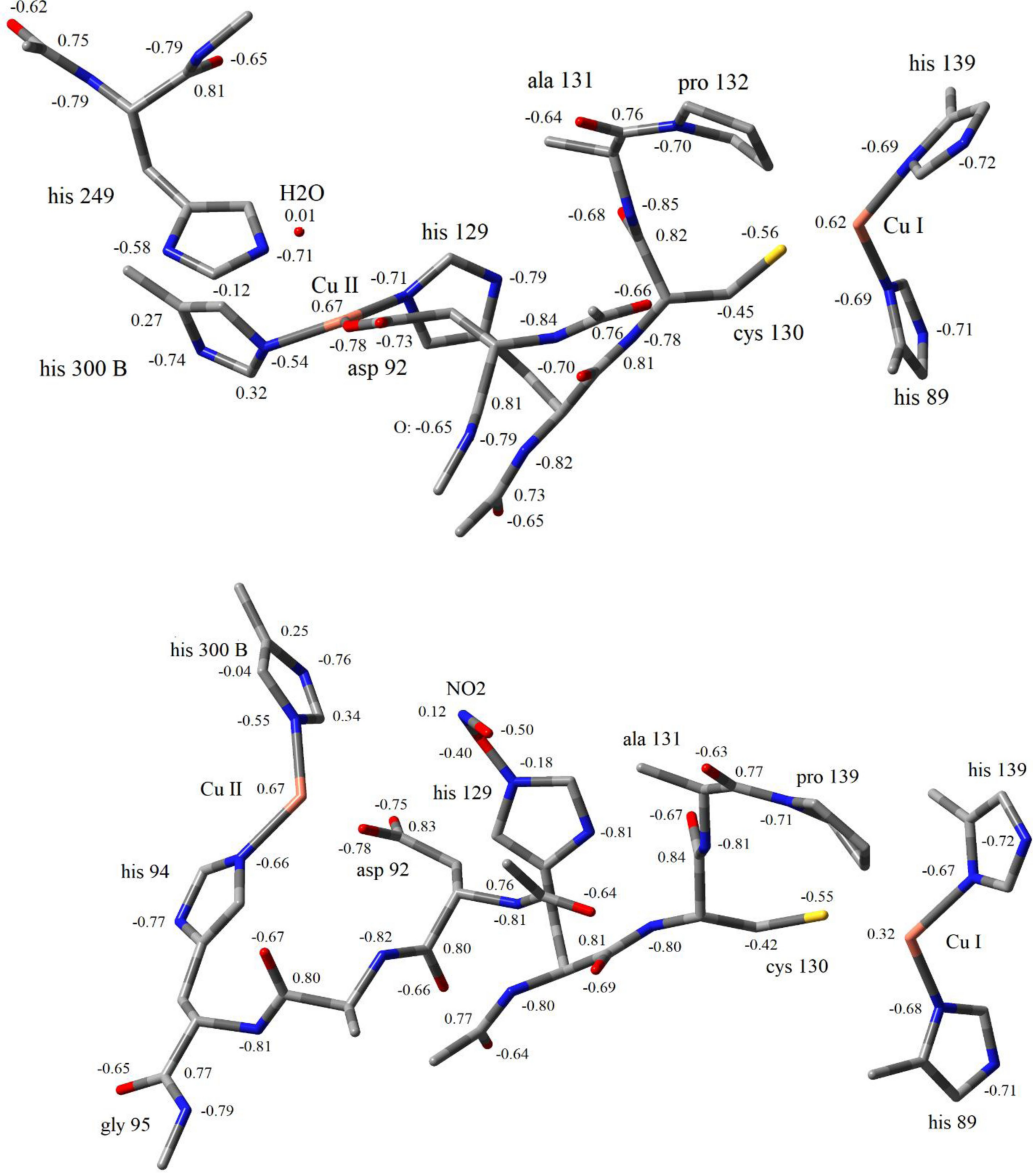
Top: Single‐atom Mulliken charges of the 150 atom CuNIR H_2_O fragment. Bottom; Single‐atom Mulliken charges of the 148 atom CuNIR NO_2_^−^ fragment. The charges shown are those on the Cu atoms, as well as all charges on the hetero atoms, N and O. Also included are selected charges on C atoms, especially those connected to O and N atoms. Charges on C and N atoms of His300 B are also included.

We finally computed the Mulliken electrostatic charges^[34]^ of all the atoms in the 150 and 148 atom CuNIR fragments, Figure 9. Although pronounced structural and electronic changes accompany the H_2_O→NO_2_^−^ substitution, only less than a tenth of an electronic charge in the peptide frame can be detected. The Type I Cu‐atom is an exception, with charge redistributions in the range of >0.3 e electronic charge units. The Type I centre exhibits the largest change (decrease) of 0.3 e, even though primary conformational changes are triggered from the Type II centre. This is another reflection of the “protein quake” notion. Charge transfer appears to be between the Type I electron inlet site and bound nitrite, with no excess charge residing temporarily at the Type II Cu centre. The charge redistribution can be compared with NMR spectroscopy data for the paramagnetic (oxidized) form of the blue copper ET protein plastocyanin,^[35]^ which shows that excess electronic ***spin*** density expands much further through the protein structure than excess electronic ***charge*** density. Even though small, the Mulliken charge redistribution is thus another reflection of the conformational “protein quake” notion.

## 6 Some Notes of Summary and Perspectives

Our study is prompted by observations that multicentre redox metallenzymes, blue copper oxidases in particular, often show no voltammetry of their own, whereas metalloenzyme/‐protein immobilization on electrochemical surfaces or on binding of enzyme substrate molecules trigger drastic intramolecular and interfacial ET changes.[^3^, ^5^, ^6^, ^12^] Cyt *c*_4_ and the blue Cu‐enzymes CuNIR and laccase are paradigmatic cases.[^5^, ^10^, ^25^, ^28^] These observations apply both to macroscopic voltammetry (at well‐defined single‐crystal SAM modified electrode surfaces) and to the level of the single molecule in electrochemical *in situ* STM and AFM. Our present focus is the blue copper enzyme copper nitrite reductase, CuNIR (*A. xylosoxidans*, PDB 1OE1). Each subunit in the trimeric enzyme is a ***two***‐centre metalloenzyme with a Type I Cu centre for electron inlet and a Type II catalytic Cu centre for nitrite reduction. Molecular scale mapping of microscopic redox potentials and ET rate constants for ***two‐centre*** proteins is within reach,[^3^, ^5^, ^6^] whereas microscopic ET and ligand binding networks in four‐centre metalloproteins such as cyt *c*_3_^1^ or hemoglobin^[2]^ are far too complex for complete microscopic resolution.

We have specifically addressed, ***why*** NO_2_^−^ binding to CuNIR may trigger not only strong voltammetric signals, but also substantial structural expansion (“swelling”) of electrochemically immobilized CuNIR, and at the same time strong single‐molecule *in situ* STM contrasts. The latter reflects high electronic single‐molecule conductivity of the NO_2_^−^ bound active enzyme as opposed to electrochemically inactive enzyme in the absence of nitrite. With a view on these apparently counterintuitive observations, we have reported a detailed theoretical and computational study of the CuNIR H_2_O→NO_2_^−^ substitution that follows a recently reported preliminary study of ours.^[3]^ Our study rests on a strategically chosen 740 atom model monomeric *A. xylosoxidans* CuNIR fragment that envelopes the two Cu‐centres and their ligand spheres. A first major outcome is that protein “swelling” as observed by electrochemical AFM,[^3^, ^28^] and >2 Å Cu−Cu distance expansion indeed accompanies H_2_O→NO_2_^−^ substitution.^[3]^ At the same time this ***nuclear*** conformational reorganization takes the frontier LUMOs and HOMOs from being through‐space poorly conducting to energetically closely aligned and spatially highly delocalized.

Here we have analyzed and consolidated the computed conformational changes and MO delocalization in much greater detail than in our first study. We have identified in detail the dynamics of the individual amino acid residues that dominate the conformational changes in the 740 atom CuNIR fragment and recalculated the changes in the electronic densities in the frontier orbitals HOMO and LUMO on H_2_O→NO_2_^−^ substitution. The conformational reorganization is triggered around the Type II Cu centre but transmits into the region between the two Cu‐centres. A second Cu−Cu channel involving Ala131 may also open. The specific outcomes of our present study can be summarized as:

– Only little electrostatic (Mulliken) atomic charge^[34]^ redistribution in the peptide frame occurs. The electrostatic charge changes of the Type I Cu centre exhibits the largest change of 0.3 e, even though the conformational changes are triggered from the Type II Cu centre. Seemingly the electron transferred does not enter a Type II Cu‐orbital, but is delocalized via the Type II Cu His129 ligand to bound nitrite, by superexchange or even two‐step “hopping”,^[36]^ possibly accompanied by proton transfer in a PCET mode, Equation (1).[^12^, ^36^] This pattern is a first reflection of the “protein quake” notion through the protein in NO_2_^−^ binding.
– The major conformational switching triggered by H_2_O→NO_2_^−^ substitution involves several Type II Cu ligands and is prompted, first by breaking of the H_2_O ligand H‐bonds to His300 and His129 Type II Cu ligands, “loosening” the whole Type II Cu ligand structure. This causes both a pronounced shortening of the His94 Cu−N bond from a loose 5.53 Å interaction in the H_2_O fragment to a 2.05 Å coordinative bond in the NO_2_^−^ fragment, and opposite, virtual dissociation of the Type II Cu His129 Cu−N bond from 2.04 Å to 5.33 Å. Both these major structural changes contribute to the observed enzyme structural expansion.
– Within the fragment model used, bound NO_2_^−^ is “associated” with the Type II Cu His129 ligand, rather than coordinatively bound to Type II Cu as in the crystal structure (*Rhodobacter sphaeroides* CuNIR PDB 1WA1 and 1WA2).[^17^, ^18^, ^19^, ^20^] Structural loosening and expansion on H_2_O liberation is not compensated by NO_2_^−^ coordination. As noted, this pattern differs from the small crystallographic differences which, as also noted is not the natural (electro)catalytic medium. Type I Cu ligand nuclear reorganization is smaller. This is notable, as the Type I Cu Mulliken charges undergo the more significant changes but accords with the small structural changes that follow broadly ET in blue Cu‐protein Type I Cu centres.
– The carboxylate Type II Cu/Asp92 coordination is significantly tightened on H_2_O→NO_2_^−^ substitution, from loosely bound H_2_O (2.01/4.16 Å) to tightly NO_2_^−^ bound (2.27/2.24 Å). This structural triggering is opposite to the loosening of the Type II Cu/His129 coordinative bond (2.04→5.33 Å).
– The replacement of the H_2_O H‐bond link(s) in the superexchange pathway by the largely through‐space link on NO_2_^−^ “association” would induce structural “swelling” expected to attenuate the electronic contact between the Type I and Type II Cu centres. “Swelling” (conformational expansion) is, however, accompanied by drastic electronic delocalization of the frontier MOs. From being largely localized around the Type I Cu centre in the H_2_O fragment, and with a wide and poorly conducting space to the Type II Cu centre, NO_2_^−^ binding delocalizes the frontier LUMO and HOMO to the whole region between the Type I and Type II Cu centres. The delocalization also involves close electronic contact‐between NO_2_^−^ and the now displaced Type II Cu His129 ligand, closing the gap between His129 and Type II Cu. The conformational reorganization (“quakes”) triggered by NO_2_^−^ binding may also bring in electronic ET contribution from Ala131 via an Ala131/His129 hydrogen bond ending at the Asp92 carboxylate Type II Cu ligand. The major Asp92 “swing” from (2.01/4.16 Å) to tightly bound (2.27/2.24 Å) on H_2_O→NO_2_^−^ substitution thus triggers conformational changes around Type II Cu that transmit conformationally and electronically through much of the protein.

As an epilogue, condensed matter chemical and biological charge transfer theory offers the following overarching form of the rate constant, W (s^−1^):[^8^, ^10^]

(2) $$W=\kappa_{el}\frac{\omega_{eff}}{2\pi }\exp\left(-\frac{\Delta G^{\neq }}{k_{B}T}\right)$$

*ω*_eff_ is a vibrational frequency averaged over all the protein, solvent, and low‐frequency intramolecular nuclear modes. $\Delta G^{\neq }$ is the activation (free) energy holding the ***nuclear*** reorganization (free) energy, driving force, gating etc. In our study we have focused on the ***electronic*** transmission coefficient, *κ*_el_, with purely ***electronic*** information about wave functions, and spin and distance effects. As noted, the electronic effects in the CuNIR catalytic ET process are, however, also strongly controlled by nuclear conformational gating. Forthcoming theoretical and computational efforts in mapping of the CuNIR H_2_O→NO_2_^−^ substitution could therefore address protein and solvent dynamics of CuNIR and other multicentre metalloenzymes in enzymatic action.[^8^, ^10^, ^12^]

We have, finally noted reservations regarding the CuNIR model fragment size. However, a major part of the CuNIR fragment is still intimately involved in the conformationally and electronically triggered transition from largely inactive voltammetry and *in situ* STM/AFM of the H_2_O fragment to the electrocatalytic and electronically conducting, structurally expanded state of the NO_2_^−^ fragment.

## Conflict of Interests

The authors declare no conflict of interest.

7

## Data Availability

The data that support the findings of this study are available from the corresponding author upon reasonable request.
